# Predicting postoperative facial swelling following impacted mandibular third molars extraction by using artificial neural networks evaluation

**DOI:** 10.1038/s41598-018-29934-1

**Published:** 2018-08-16

**Authors:** Wei Zhang, Jun Li, Zu-Bing Li, Zhi Li

**Affiliations:** 1 The State Key Laboratory Breeding Base of basic science of Stomatology (Hubei-MOST) and Key Laboratory for Oral Biomedical Engineering of the Ministry of Education (Wuhan University), Wuhan, China; 2 Department of Endodontics, School and Hospital of Stomatology, Wuhan University, Wuhan, China; 3 School of Mechanical and Materials Engineering, Jiujiang University, Jiujiang, China; 4 Department of Oral and Maxillofacial Surgery, School and Hospital of Stomatology, Wuhan University, Wuhan, China

## Abstract

Patients’ postoperative facial swelling following third molars extraction may have both biological impacts and social impacts. The purpose of this study was to evaluate the accuracy of artificial neural networks in the prediction of the postoperative facial swelling following the impacted mandibular third molars extraction. The improved conjugate grads BP algorithm combining with adaptive BP algorithm and conjugate gradient BP algorithm together was used. In this neural networks model, the functional projective relationship was established among patient’s personal factors, anatomy factors of third molars and factors of surgical procedure to facial swelling following impacted mandibular third molars extraction. This neural networks model was trained and tested based on the data from 400 patients, in which 300 patients were made as the training samples, and another100 patients were assigned as the test samples. The improved conjugate grads BP algorithm was able to not only avoid the problem of local minimum effectively, but also improve the networks training speed greatly. 5-fold cross-validation was used to get a better sense of the predictive accuracy of the neural network and early stopping was used to improve generalization. The accuracy of this model was 98.00% for the prediction of facial swelling following impacted mandibular third molars extraction. This artificial intelligence model is approved as an accurate method for prediction of the facial swelling following impacted mandibular third molars extraction.

## Introduction

The extraction of impacted mandibular third molars is one of the most common surgical events. Patients’ postoperative facial swelling following third molars extraction may have both biological impacts and social impacts. The severity of the facial swelling is depending on the case and the facial swelling originated in an inflammatory process that initiated by surgical trauma. The facial swelling is more likely occurring in those patients who received impacted mandibular third molars extraction with utilizing flap operation and bone removal. The factors thought to influence the incidence of facial swelling after third molar removal include patients’ age, gender, physique and oral hygiene^1–5^. In addition, the incidence of facial swelling is related to the type of third molar, the degree of impaction, and ease of extraction operations^1–8^. As far as a specific patient is concerned, most of oral surgeons could predict the incidence of the facial swelling solely based on their personal experience. The first attempt to create a model of prediction was published by MacGregor^7^, who established a multivariate model based on radiographic findings. Berge and Bøe^8^ attempted to predict the extent of postoperative morbidity following third molar surgery through multiple regression analysis. Although a number of efforts at determining a model for this assessment have been made, none of them could be praised for universal reliablility^1^. Obviously, the main reason was the difficulty of quantification of the magnitude of the contributions of the different categories of variables.

Artificial nerve networks principle can be used to analyze the multiple factors and the relationships between the factors which have unclear relationships, and it is a system based on the imitation of human brain structure and function. Neural networks method is a branch of biomedical engineering known as artificial intelligence (AI). Artificial neural networks, due to their excellent ability of non-linear mapping, generalization, self-organization and self-learning, have been proved to be of widespread utility in digital signal processing, system modeling, automatic control, and others^9–12^. The applications of neural networks have received a great deal of attentions in clinical medicine. By properly choosing neural networks structures and training the weights, researchers could use neural networks to perform medical outcome prediction^13–18^. Neural networks have the capacity to “learn” how to make a diagnosis through the information presented to them^19–21^.

In this present study, an AI model was established with improved conjugate grads backpropagation (BP) algorithm to predict facial swelling following impacted mandibular third molars extraction. The software we used for the experiments is MATLAB’s Neural Network Toolbox^22^. 5-fold cross-validation is used to get a better sense of the predictive accuracy of the neural network and early stopping is used to improve generalization. The purpose of this study was to evaluate the accuracy of this improved neural networks model in predicting.

## Methods and Materials

### Study population

The study protocol was approved by the Ethics Committee of the School and Hospital of Stomatology, Wuhan University, Wuhan, China. All patients were informed of the clinical study, and they provided signed informed consent. The study was conducted in full accordance with ethical principles, including the Declaration of Helsinki. All methods were performed in accordance with the relevant guidelines and regulations. The 400 patients, who received impacted mandibular third molars extraction in Hospital of Stomatology, Wuhan University, were randomly selected in this study. There were 300 patients assigned as the training samples, which is the design set,and another 100 patients were made as the test samples. All samples were selected from a pool of patients admitted for mandibular third molars extraction between January 2014 and December 2016.

These selected patients had no known immune impairment, no contraindications for oral surgery and no medication taking. The surgical procedure was standardized and uniformly performed by one same oral surgeon, and patients strictly followed post-extraction instructions. Antibiotics were used for three days after extraction to prevent infection in all patients. A single examiner performed all clinical measurements prior to surgery as well as 72 hours after the operation.

In this study, the parameters, which related to the occurrence of facial swelling following impacted mandibular third molars extraction, were considered as the networks input, including 15 factors. These 15 factors were consisted of patient’s age, gender, physique and oral hygiene as personal factors; relation of the third molars to the mandible ramus and second molar, relative depth of the third molars in bone, relationship of the long axis of the third molars in relation to the long axis of the second molar, relation of the third molars in mandibular dental arch, number of root as anatomy factors of third molars; and type of incision, location and quantity of bone removal, section into pieces or not, root fracture condition, fracture of lingual bone plate or not, surgical time as factors of surgical procedure. All the parameters and the data normalization are shown in Table 1.

**Table 1:** Normalization of the parameters data.

Parameters	Normalization
X1: Gender	Male: 0.5; Female: 1
X2: Age	≤20: 0.5; 20–30: 0.25; 30–40: 0.5; ≥40: 1
X3: Physique	Strong: 0; Middle: 0.5; Slim: 1
X4: Oral hygiene	Good: 0; Middle: 0.5; Bad: 1
X5: Relation of the wisdom teeth to the mandible ramus and second molar	Type I : 0; Type II: 0.5; Type III: 1
X6: Relative depth of the wisdom teeth in bone	High: 0; Middle: 0.5; Low: 1
X7: Relationship of the long axis of the wisdom teeth in relation to the long axis of the second molar	Vertical: 0; Buccalclination/Lingualclination: 0.25; Mesialclination/Distalclination: 0.50; Horizontal: 0.75; Invertion: 1
X8: Relation of the wisdom teeth in mandibular dental arch	Lingual displacement: 0.25; Normotopia: 0.5; Buccal displacement: 1
X9: Number of root	1: 0; 2: 0.5; ≥3: 1
X10: Type of incision	No incision: 0; Buccal incision: 0.5; Distal incision: 0.5; Buccal incision + Distal incision: 1
X11: Location and quantity of bone removal	No: 0; Buccal/Distal/Occlusal: 0.5; Buccal + Distal/Buccal + Occlusal/Distal + Occlusa: 0.75; Buccal + Distal + Occlusal: 1
X12: Section into pieces or not	no section: 0; section into 2 pieces: 0.5; section into 3 or more pieces: 1
X13: Root fracture condition	No: 0; 1 root fracture: 0.25; 2 roots fracture: 0.5; ≥3 roots fracture: 1
X14: Fracture of lingual bone plate or not	No: 0; Yes: 1
X15: Surgical time	≤10 minutes: 0; 10–20 minutes: 0.5; ≥20 minutes: 1

According to Table 1, the parameters data of 400 samples were normalized and counted as the inputs. The patients’ prognosis data were the output, in which severe swelling was recorded 1, moderate swelling was recorded 0.5, and mild swelling was recorded 0.

### Measurement of facial swelling

The facial swelling measurements were taken using a 2–0 nylon thread and a millimeter ruler before the surgery and 72 hours after the operation. To evaluate the swelling, markings with permanent marker were made prior to the surgery on the following facial regions: the angle of the mandible, the tragus, the labial commissure, the nasal border, laterally to the external corner of the eye, and on the soft pogonion. The following measurements were taken: Distance I (from the angle of the mandible to tragus); Distance II (from the angle of the mandible to the external corner of the eye); Distance III (from the angle of the mandible to the nasal border); Distance IV (from the angle of the mandible to the labial commissure); Distance V (from the angle of the mandible to the soft pogonion)^23,24^. Differences between the measurements prior to surgery and those made 72 hours after the operation at distances were recorded, and the average of the five differences value was calculated. When the average of the five differences value was less than 10 mm, the patient was classified as mild swelling; 10–20 mm was moderate swelling; more than 20 mm was severe swelling. All postoperative data were recorded by the same independent blinded investigator in order to avoid observer bias.

### Basic Principle of the neural networks model

**Figure 1:**
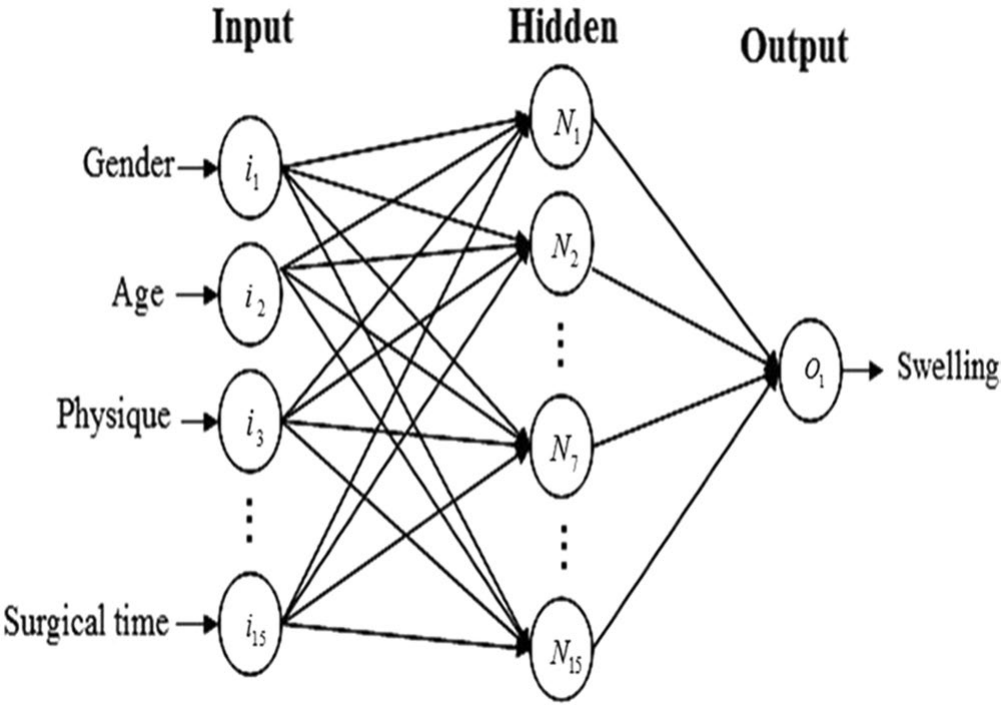
The architecture of the neural networks model.

The neural networks model based on the improved conjugate grads BP algorithm combining adaptive BP algorithm and conjugate gradient BP algorithm is shown in Fig. 1.

The neural network used in this experiment was a one-hidden-layer perception with the improved conjugate grads BP algorithm that had 15 neurons in the input layer, 15 in the hidden layer, and 1 in the output layer. Figure 1 illustrates this neural network, Where in and Nn (n varies from 1 to 15) are the n^th^ input neuron and the n^th^ hidden-layer neuron, respectively, and Ok is the output neuron for k^th^ set of input data. These artificial neurons passed information from one layer to the other, in a process that imitated a human synapse, and the processed information produced an output signal. The number of interactions or learning cycles of the network was established upon trial and error in all the interactions, and used the reduction of the mean square error (MSE) as learning criterion.

Like conventional BP algorithm, conjugate gradient BP algorithm has the same process of propagating the input forward through the networks. The main difference between them is the process of propagating the error backward through the networks. The conjugate gradient BP algorithm reduces the amount of computation in the training process, thus there is no need to calculate the search direction in every step of the iterative process in the conventional BP algorithm.

### Algorithm process

The process of the improved BP Algorithm is as follows. At the first step, the networks weights are initialized by random values, and the training sample values are inputted, in which the required error precision is set as 10^−3^. At the second step, propagating the input forward through the networks, and judging whether MSE of sample meets the required error precision (10^−3^). If it does, then the learning will end. At the third step, it is judgment that whether the networks training enters extreme point. If it does, then the conjugate gradient method will be used in the backpropagating of networks. Otherwise, the adaptive BP algorithm will be used in the backpropagating of networks. At the fourth step, they are propagating the optimized weights forward through the networks, and judging whether the MSE of sample meets the error precision. If it does not, then it will return to the third step. After the above four steps, one epoch of the improved BP Algorithm is completed, in which epoch is an iteration of subset of the samples for training algorithm.

### Judging whether the networks training enters extreme value

At the third step of the algorithm process, it is necessary to judge whether the networks training enters extreme point. In the present study, 10 epochs training is the interval, and the difference value of training errors in the intervals is evaluated. If the difference value is less than the judging value of extreme point, the networks training is considered as entering the extreme point. The judging value of extreme point will influence the speed of networks training. If the judging value of extreme point is too high, error function of the sample will not meet the conditions to further use conjugate gradient method, which will have trouble with overflow. If the judging value of extreme point is too low, conjugate gradient method will be used until networks training is trapped in the local minimum for a long time, which will then result in remarkably slow networks training speed.

## Results

### Networks training

When adaptive BP algorithm was applied, the learning curve would be apt to be trapped in infinitesimal, and there would be still no convergence until training epoch was over 100000. When conjugate gradient BP algorithm was used in networks training of the same sample, it would be apt to overflow. However, when improved BP algorithm was used, the problem of local minimum would be avoided effectively by networks training, and the speed of networks training would be improved greatly. The networks training epoch would be 13 when networks training of this sample converged to the condition that MSE was 0.001.

### Judging value of extreme point has influence on networks training speed

**Figure 2:**
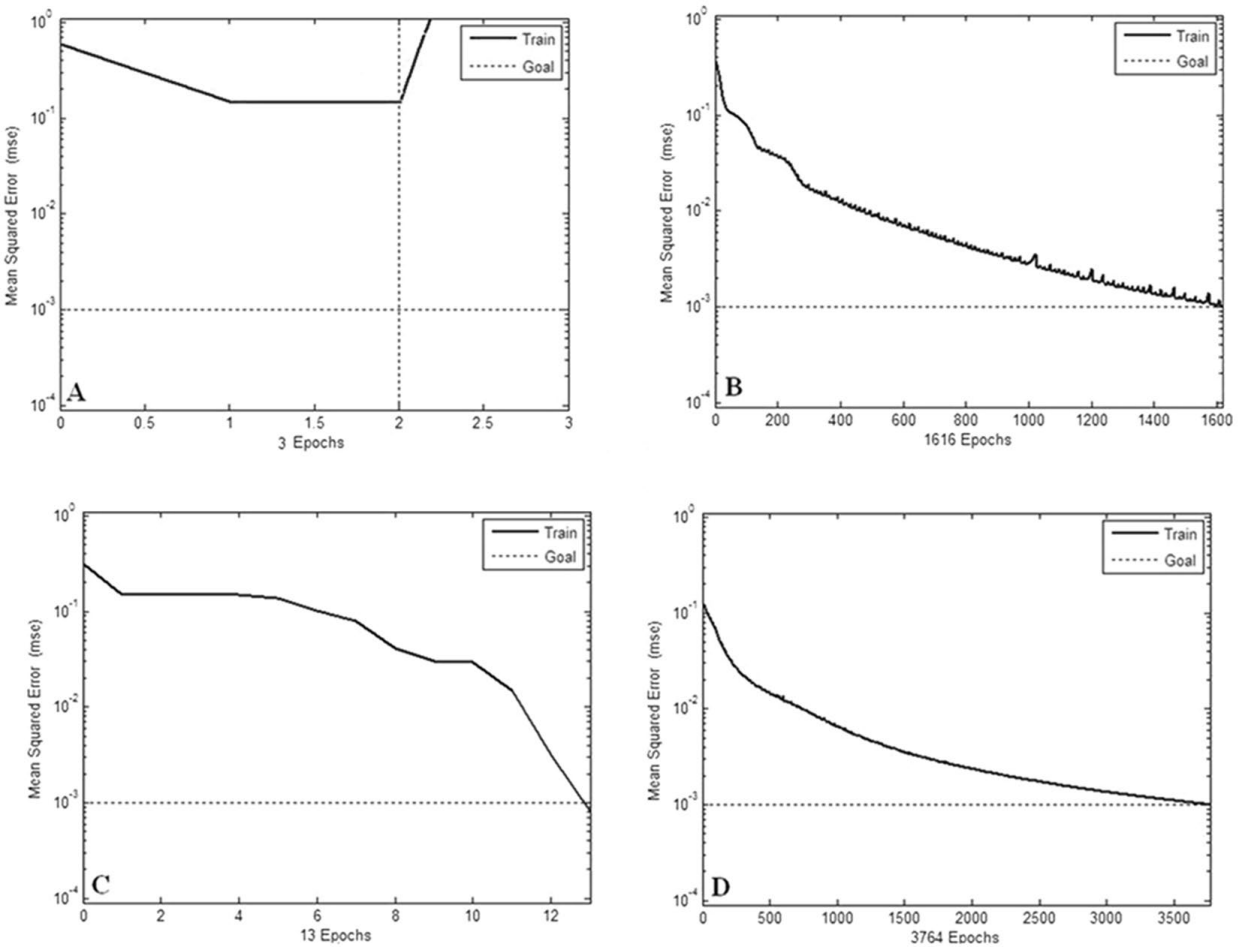
(**A–D**) The different networks training epochs under four different judging value of extreme point (A: 0.1; B: 0.01; C: 0.001; D: 0.0001.).

When the required error precision was set at 10^−3^, networks training epochs were represented as different judging values of extreme point (Fig. 2A–D). From the Fig. 2, we can obtain the Table 2, which shows the convergence epoch of networks training when different judging values of extreme point were set. When judging value of extreme point was set at 0.001, networks training speed would be the fastest.

**Table 2:** Judging value of extreme point has influence on networks training speed.

Judging values of extreme point	> 0.1	0.01	0.001	0.0001
Training epochs	overflow	1616	13	3764

### Improving the predictive accuracy of the neural network and improving generalization

To get a better sense of the predictive accuracy and improve generalization, 5-fold cross-validation and early stopping were used. In this technique the available data (400 samples) is divided into two subsets. the first subset (300 samples) is the designing dataset, which is used for training and validating the neural network, the second subset (100 samples) is the test dataset.

5-fold cross-validation and early stopping technique were used in order to get a better sense of the predictive accuracy and avoid overfitting. 5-fold cross-validation splits the designing dataset into five parts at random. It trains five new neural networks, each one on four parts of the designing dataset, it then examines the predictive accuracy of each new neural network on the data not included in training dataset, which is named the validation set. The method for improving generalization is called early stopping. In this technique, the error on the validation set is monitored during the training process. The validation error normally decreases during the initial phase of training, as does the training set error. However, when the network begins to overfit the data, the error on the validation set typically begins to rise. When the validation error increases for a specified number of iterations, the training is stopped, and the weights and biases at the minimum of the validation error are returned. In this case, every training fold contains roughly 4/5 of the designing dataset and every validation fold contains roughly 1/5 of the designing dataset.

The test dataset which is not used during training, but it is used to compare different models.

### Results of prediction test

**Figure 3:**
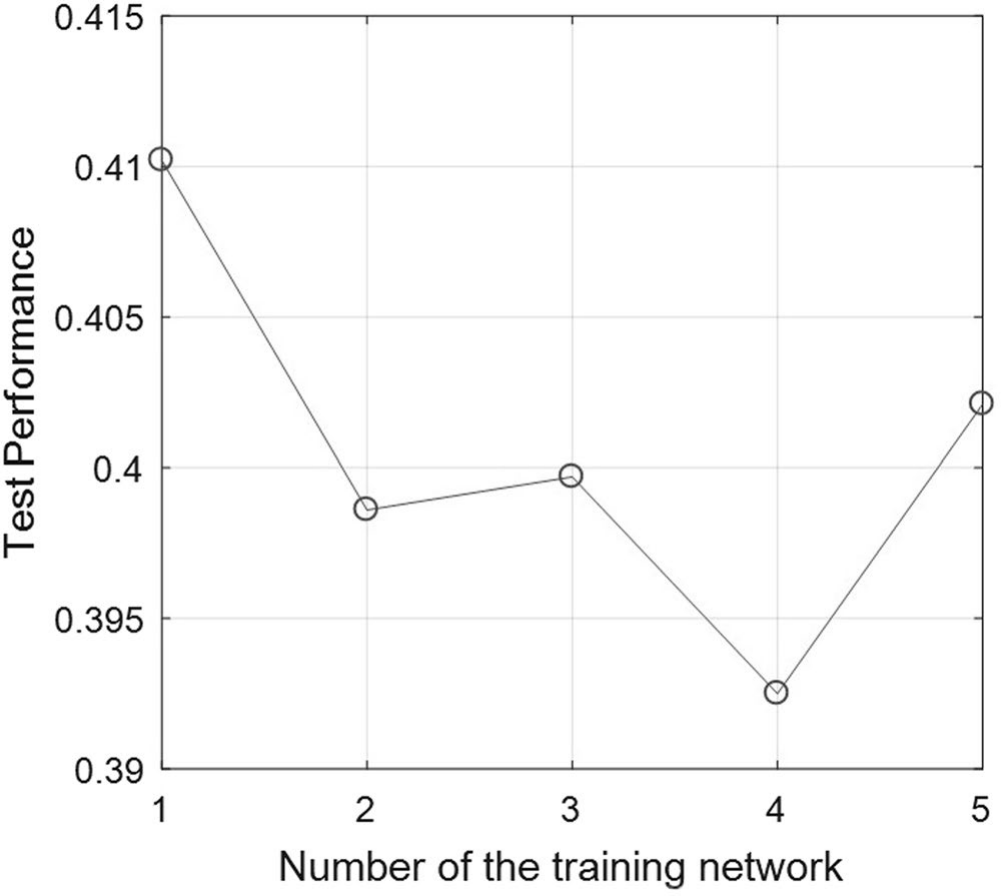
The test performance of the training network in 5-fold cross-validation.

When the neural network output value was lower than 0.4, it meant mild or no swelling; when it was higher than 0.6, it meant severe swelling; when it was between 0.4 and 0.6, it meant moderate swelling. When 5-fold cross-validation and early stopping technique are used, the test performance of each training network in 5-fold cross-validation is showed in Fig. 3. The best test performance is the fourth time of cross-validation, its value is 0.3925, which was computed using matlab’s function perform^22^.

**Figure 4:**
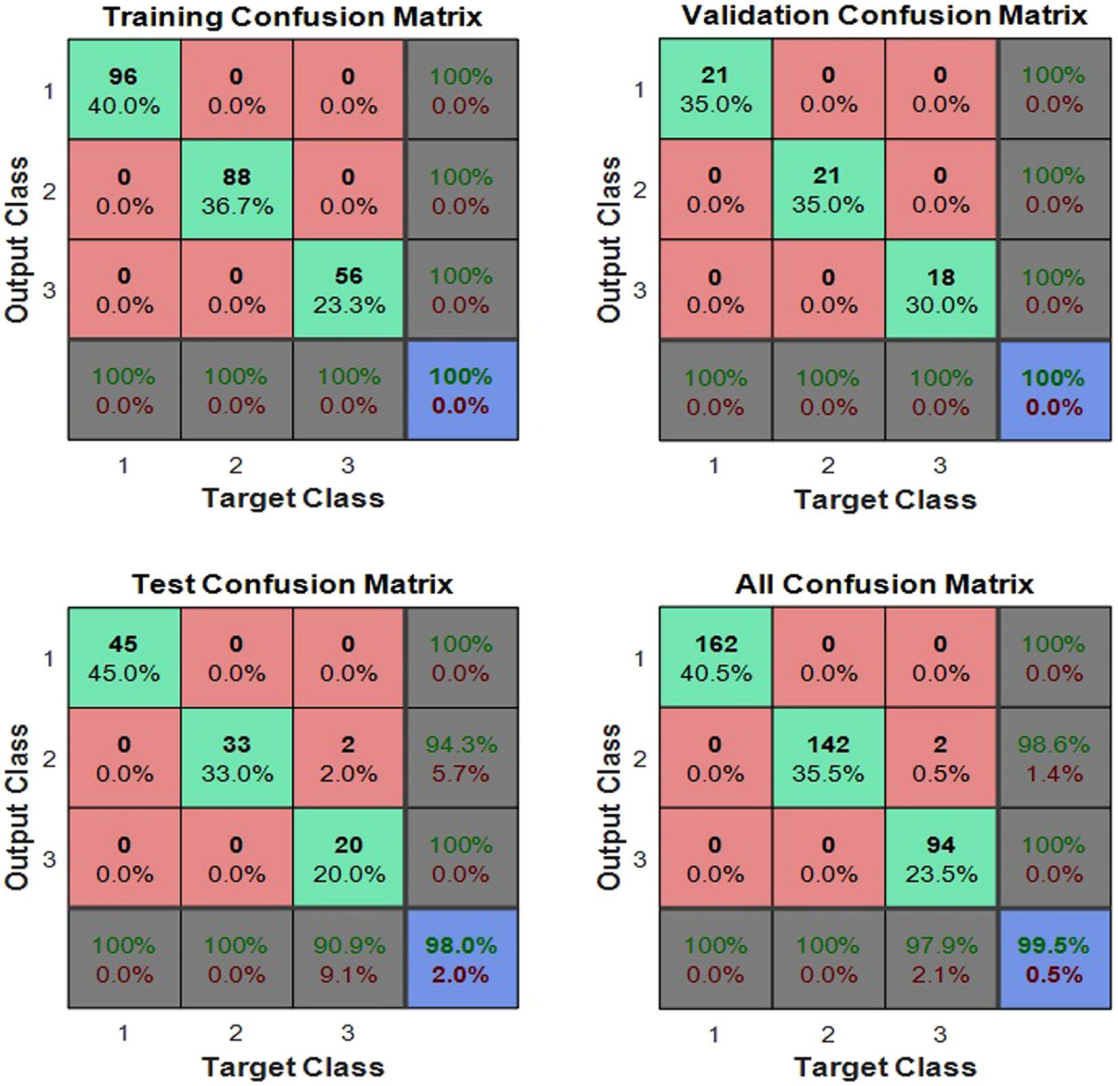
The confusion matrix of the dataset.

**Figure 5:**
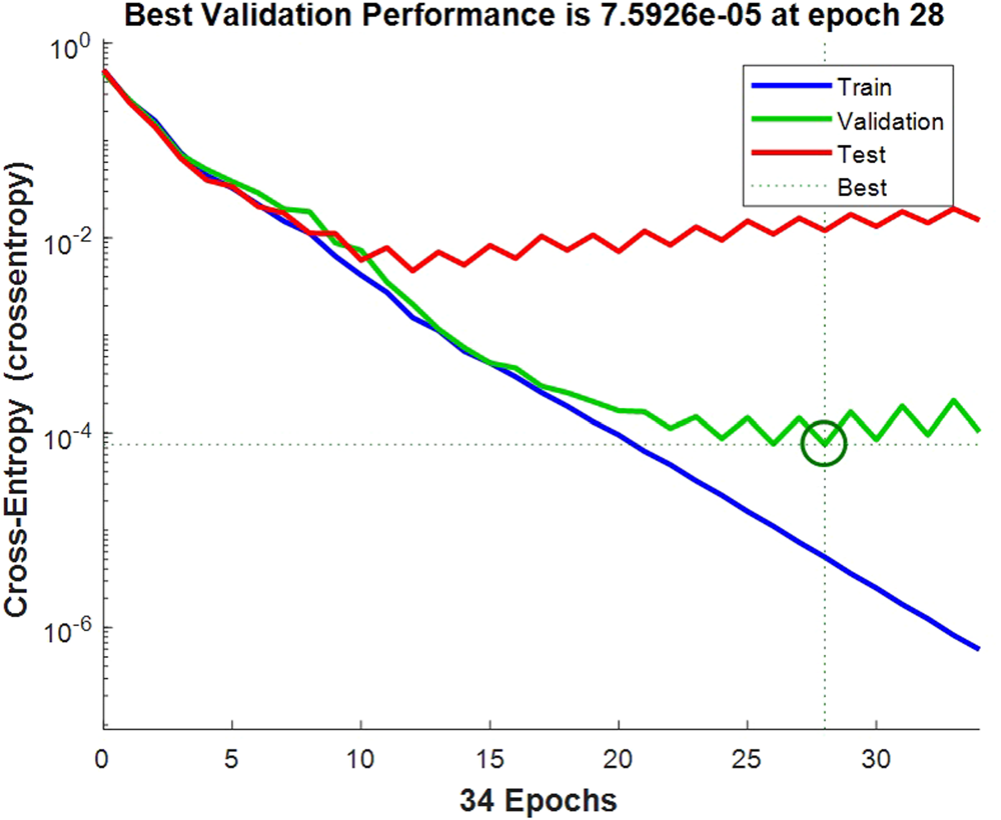
The error vs. epoch for the training, validation, and test performances of the training record.

**Figure 6:**
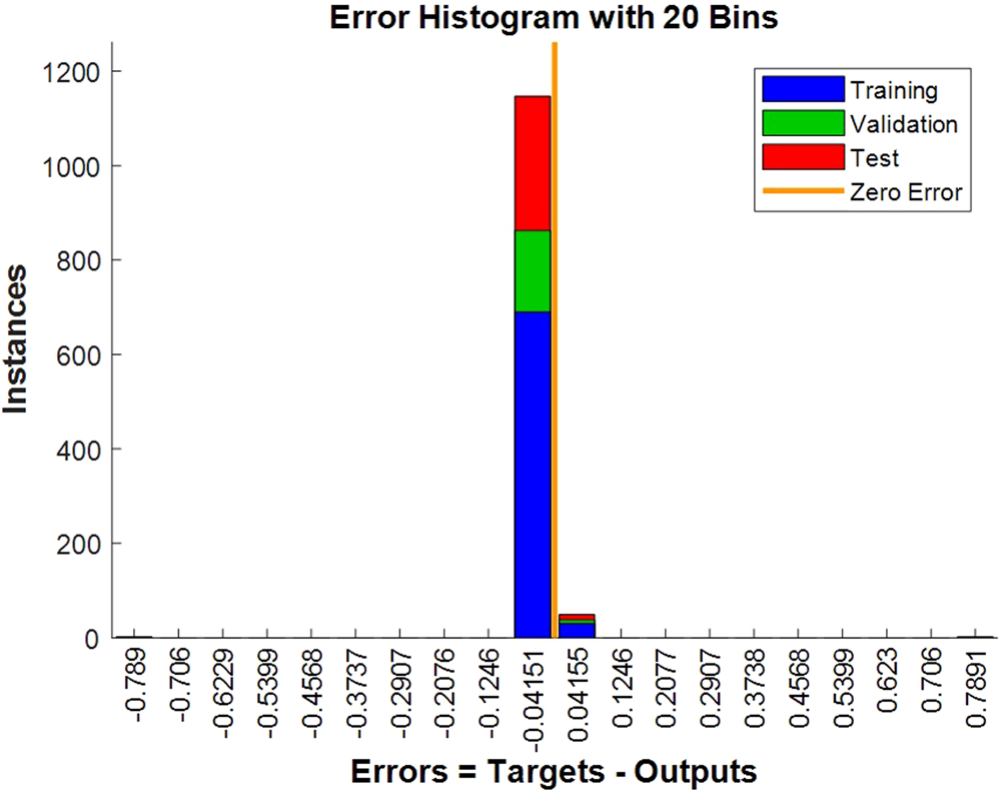
The error histogram.

The test dataset (the diagnosis 100 samples) was performed with prediction test by the fourth trained network of 5-fold cross-validation. The confusion matrix of the test dataset, the validation dataset, the test dataset and all of the dataset was shown in Fig. 4, the test dataset and the validation dataset are both fitted, while two cases misfit in the test dataset. The network’s performance was measured according to the mean of squared errors, error vs. epoch for the training, validation, and test performances of the training record was shown in Fig. 5, the training stops after 28 consecutive increases in validation error, and the best performance is taken from the epoch with the lowest validation error. Error histogram was shown in Fig. 6. As is shown in Table 3, in the prediction test dataset, 98 cases did match the reality, and the prediction accuracy was 98.00%.

**Table 3:** Results of prediction test.

The output values of the network	< 0.4 (mild or no swelling)	0.4–0.6 (moderate swelling)	> 0.6 (severe swelling)	Total
The number of the reality	35	47	16	98
The number of correct prediction	33	46	15	94

## Discussion

Postoperative facial swelling is a common complaint after impacted third molar removal, and the swelling degree is different among the patients. The swelling differs depending on the patients’ characteristics, preoperative difficulty index, and intraoperative factors^1–8^. All clinicians stress the need for swelling control in patients who undergo third molar surgery, and many investigations focused on reduction of the swelling^23–27^. However, there is no special intelligent method to predict the swelling. The inaccuracy in the prediction of the swelling response always affects the quality of medical services and the follow-up treatment plan.

The factors that contribute toward the postoperative facial swelling following third molar surgery are sophisticated. In this study, 15 variables were recorded as the parameters in the artificial neural networks. The major advantage of artificial neural networks is their ability to synthesize a considerable amount of factors (or variables) without requiring statistical modeling of the problem. In addition, artificial neural networks learn to recognize patterns from entered data through training. Once it has been trained on entered data, the network is able to recognize similarities when presented with a new input pattern, which will result in a predicted output pattern^28^.

It is worth mentioning that we have employed computerized system based on artificial neural networks to predict the swelling following impacted mandibular third molars extraction, which shows satisfying results. The improved conjugate grads BP algorithm combining adaptive BP algorithm and conjugate gradient BP algorithm together can converge to a global optimal solution in certain conditions, which not only avoid the problem of local minimum effectively, but also improve networks training speed greatly^29^.

The ‘feed-forward, back propagation’ neural networks usually trained by BP of errors is perhaps the most popular networks architecture today^30^, in which the neural networks based on adaptive BP algorithm is a new widely used diagnosis and prediction method. Adaptive BP algorithm, however, may generally be trapped in a local minimum. Another variation on BP algorithm, the conjugate gradient BP algorithm allows the searching the direction of deterioration as a certain probability in each step, because each iteration is not a single negative gradient direction but a conjugate direction, Hence, conjugate gradient BP algorithm can converge to a global optimal solution in certain conditions, which can solve the local minimum problem of adaptive BP algorithm. However, conjugate gradient method requires the error function is convex, and the direction of each iteration requires exact linear search. The practical problems often can not meet the conditions. In general, error function of extreme value shows characteristics of convex function, and the conjugate gradient method being applied near the extreme value will improve convergence speed and learning accuracy. Therefore, the algorithm combining adaptive BP algorithm and conjugate gradient BP algorithm together may be an ideal neural networks model, which is able not only to avoid the problem of local minimum effectively, but also to improve networks training speed greatly. We believe this improved conjugate grads BP algorithm could serve as a valid neural networks model to predict the swelling following impacted mandibular third molars extraction. In this present study, the improved algorithm combining adaptive BP algorithm and conjugate gradient BP algorithm is used to establish a valid neural networks model applied in the prediction of facial swelling following impacted mandibular third molars extraction.

In this study, 5-fold cross-validation and early stopping were used to get a better sense of the predictive accuracy and improve generalization. The prediction accuracy of this method is 98.00%, which can meet the needs of actual diagnosis or prediction in clinic. There are two reasons may explain the fact that the prediction unable to achieve higher accuracy. First, there are too many related parameters (n = 15) involved in this study. In the follow-up study, some minor parameters should be abandoned, in order to optimize the networks training and improve the prediction accuracy. Second, the normalization process of these parameters data is lack of objective evidence. In future, the mathematical formulae of normalization for these parameters data should be sought to improve the scientificity and accuracy of sample.

In conclusion, this artificial neural networks based on the improved conjugate grads BP algorithm has a high prediction accuracy, which is a new developed system that can help to predict the swelling following impacted mandibular third molars extraction.
